# Simple solutions to false results with plate/slide agglutination tests in diagnosis of infectious diseases of man and animals

**DOI:** 10.1016/j.mex.2015.08.001

**Published:** 2015-09-05

**Authors:** Hari Mohan Saxena, Shubhada Chothe, Paviter Kaur

**Affiliations:** Department of Veterinary Microbiology, College of Veterinary Science, Guru Angad Dev, Veterinary and Animal Sciences University (GADVASU), Ludhiana 141004, India

**Keywords:** Superagglutination test, Superagglutination, Rose Bengal Plate Test, False positive, False negative, Agglutination test, Coombs’ test

## Abstract

We have developed a new Superagglutination test for serodiagnosis of infectious diseases. It differs from conventional plate/slide agglutination tests (PAT/SAT) by three additional steps: prior staining of serum antibody by adding a dye and addition of diluted biotinylated antiglobulin and avidin in sequence after mixing the antigen with the test serum. The new steps circumvent the problems of false positive and false negative results of PAT/SAT. In serodiagnosis of brucellosis, Superagglutination test had higher positive predictive value and specificity than Rose Bengal Plate Test (RBPT) and Standard Tube Agglutination Test (STAT) and higher negative predictive value and sensitivity than RBPT, STAT, ELISA and Complement Fixation Test (CFT).

• Superagglutination is a simple, accurate and economic screening test for infections.
• More specificity, sensitivity, positive & negative predictive value than RBPT, STAT.
• More sensitivity, negative predictive value than ELISA and Complement Fixation Test.

## Graphical abstract

**Figure:**
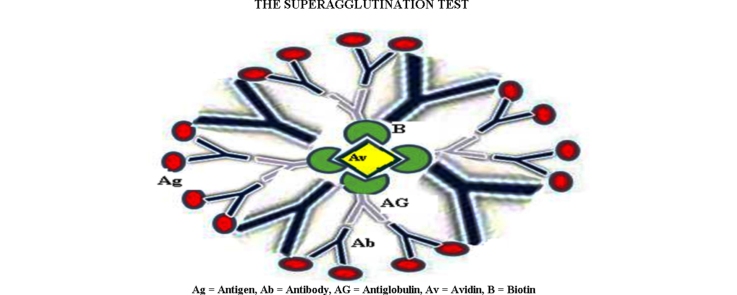

## Background

In many countries, the standard Plate Agglutination Test is the routine screening test for human and animal brucellosis. RBPT is a variant of plate/slide agglutination test where killed *Brucella* organisms stained with Rose Bengal dye are used as antigen for detection of antibodies in the serum. The RBPT is a quick, cheap and effective test for the diagnosis of brucellosis. However, it may give false negative results [1], [2]. Many factors affect the RBPT reactions and their reading. Some people are able to see the finer agglutination while many others cannot. This causes variation in results. Although the International Office of Epizootics has recommended the RBPT as one of the tests for the diagnosis of bovine brucellosis [3], some authors [4] have reported unacceptable rate of false negatives with the RBPT. Very low concentration of antibodies may not give visible agglutination. False positive results may arise due to the inability to differentiate non-specific aggregates of antigen particles alone from the true agglutinates comprising both antigen and antibody. False negative results may be due to a small clump size in sera with low titers of antibodies. False negative reactions are believed to occur mostly due to prozoning. The lack of agglutination at high concentrations of antigen or antibodies is called the Prozone effect. In Prozone, very small complexes are formed that do not clump to form visible agglutination. Prozoning may often lead to a false negative reaction in RBPT when sera of high antibody titers are tested against it. It has been suggested [5], [6] that in order to get a better diagnosis of *Brucella* infection, a combination of RBPT and ELISA should be used, especially in case of samples found negative by either RBPT or STAT used alone or in combination.

## Method details

Guidelines of the Institutional Animal Ethics Committee were followed in the study. Cattle and buffalo serum samples were derived from the animals in veterinary clinics, dairy farms and animal shelters in and around Ludhiana. All the animals were of age two years or more. Brucellosis suspected herds were selected for sampling primarily based on the history of abortions in the herd while normal healthy animals were sampled from the herds of the university dairy farm without the history of abortions and with repeatedly Rose Bengal Plate Test (RBPT) negative status. The new Superagglutination test and common serological tests i.e. the RBPT, STAT, ELISA and CFT were applied on all the serum samples (Table 1).

In the conventional RBPT, equal volumes (5 μl of each) of RBPT colored antigen (IVRI, Izatnagar, India) and test serum are mixed on a clean glass slide with the help of a clean sterilized toothpick. The slide is observed after 2 min for the formation of clumps. The formation of clear clumps is considered a positive test while the absence of clear clumps is considered a negative reaction. However, we modified the RBPT by incorporating the following additional steps in the RBPT. The modified RBPT as given below is named as the Superagglutination test [7], [8].

**Fig. 1:**
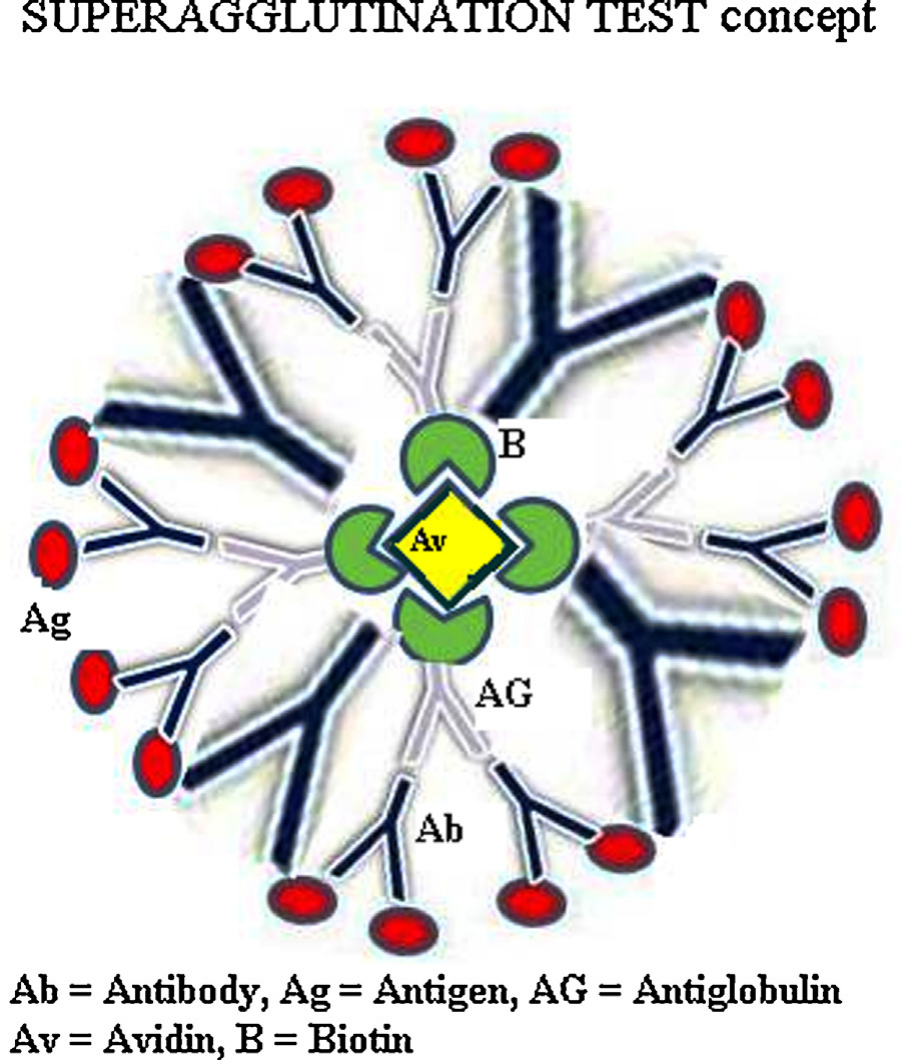
Superagglutination of antigen (Ag) and antibody (Ab) complexes by biotinylated antiglobulin (bAG) and avidin (Av).

For performing the Superagglutination test (Fig. 1), equal volumes (2.5 μl each) of RBPT colored antigen, test serum stained with 0.1% Coomassie Blue dye, biotinylated anti-bovine IgG (Sigma) and streptavidin (Sigma) were mixed thoroughly on a clean glass slide in the above mentioned sequence. The slide was observed for 4 min for the formation of clumps. Ordinary hand lens was used occasionally for better resolution. The slides were viewed under low power (10×) of an inverted microscope to visualize the composition of clumps in case of doubt. Formation of clear agglutination, within which both, the blue color and the pink color could be differentiated on magnification, were considered as positive, while absence of clear agglutinates, and aggregates of pink color alone or blue colored mass alone were considered as negative. The Superagglutination test gave superior results in detecting anti-*Brucella* antibodies compared to the other serodiagnostic tests (Table 2).

In the Superagglutination test, the test serum or plasma antibodies are mixed with a protein stain of contrasting color (like Coomassie Blue or Amido Black) to stain the antibodies. Biotinylated anti-bovine IgG and streptavidin are added to the mixture of antigen and antibodies to enhance the clump size by cross-linking the antibody molecules. Since Avidin has a strong affinity for Biotin, it will cross-link biotinylated antiglobulin bound to the antigen–antibody clumps making larger and more compact masses of clumps (Fig. 1). The additional steps of staining the test antibody and adding biotinylated antiglobulin and Avidin are our novel modifications to the conventional method of slide/plate agglutination tests. If visible clumps are formed, the test sample is positive for the antibody against the microbial antigen. In antibody control (i.e., antigen, negative serum and species-specific antiglobulin), there will be no agglutination of antigen particles. In antiglobulin control (i.e., antigen, positive serum and unrelated antiglobulin), there will be normal agglutination but no enhancement of agglutination.

## Method validation

**Fig. 2:**
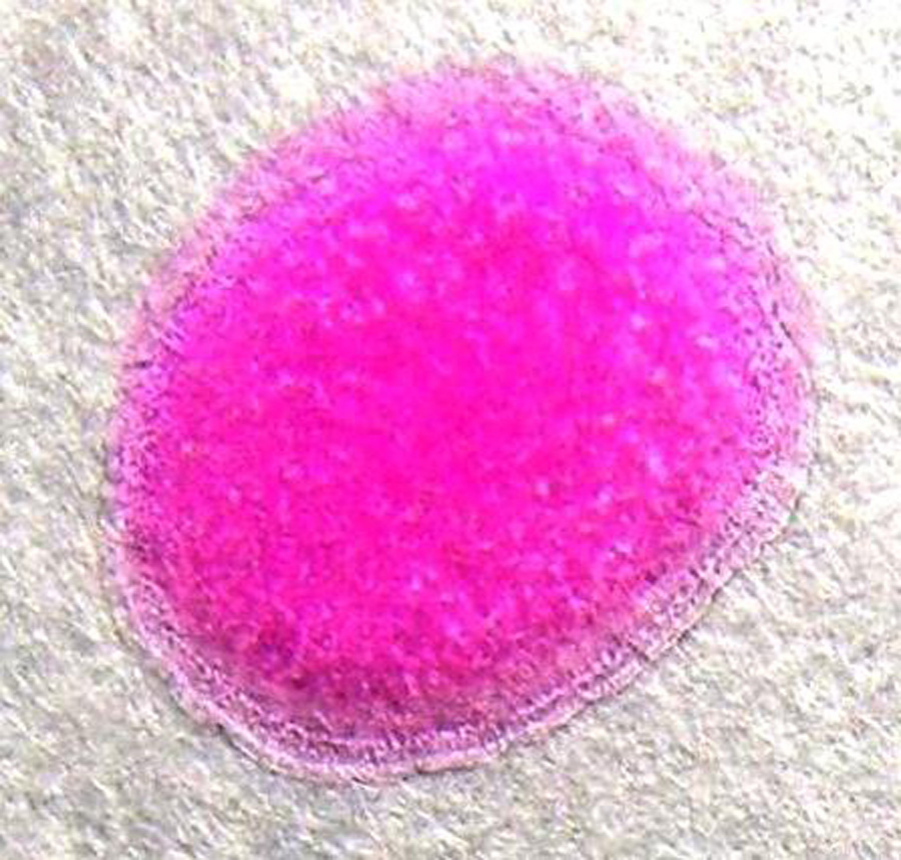
Gross view of the slide in RBPT.

The RBPT (Fig. 2, Fig. 4) detected 6% less positive samples than the Superagglutination test and showed a sensitivity of 93.33%, a specificity of 88.18%, a PPV of 86.6% and NPV of 94.17%, respectively. STAT detected 6% less positive samples than the Superagglutination test and showed a sensitivity of 94.25% and a specificity of 68.14%. PPV of this test was found to be 69.49% and NPV was 93.90%. ELISA detected 16.5% less positive samples than the Superagglutination test. Sensitivity of this test was calculated to be 74.47% and the specificity was found to be 95.24%. PPV of this test was 93.33% and NPV was found to be 80.65%. The CFT detected 14.5% less positive samples than the Superagglutination test and showed a sensitivity of 82.8% and a specificity of 93.46%.

**Fig. 3:**
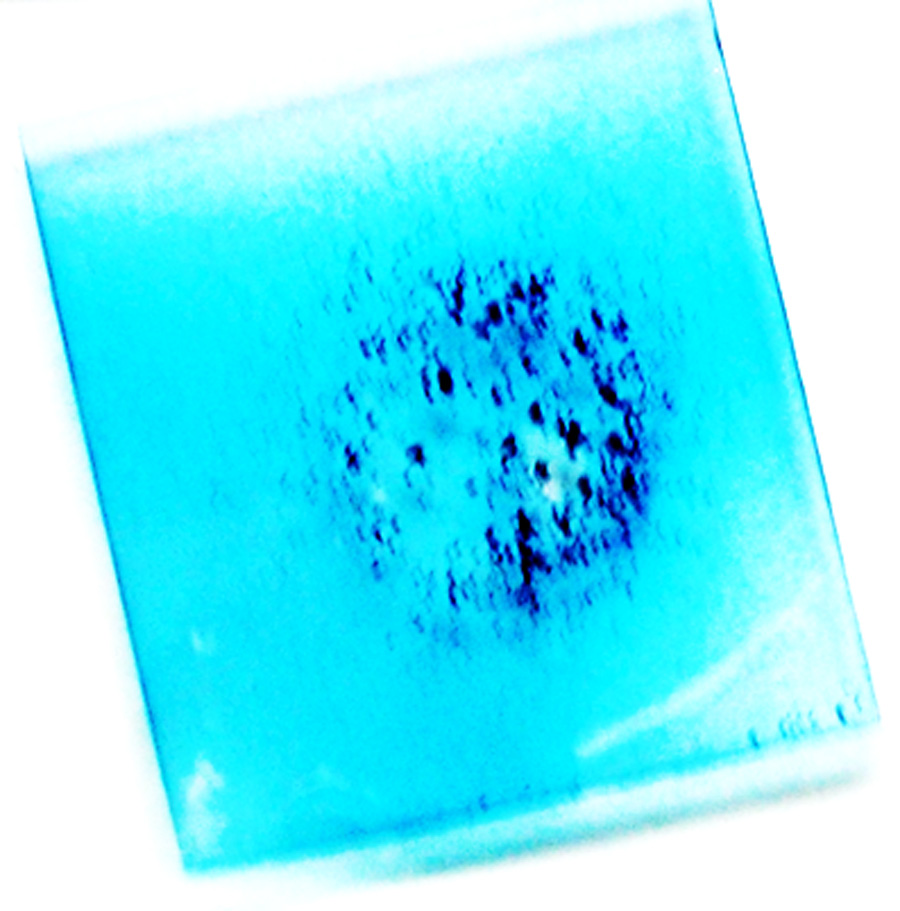
Gross view of the slide by unaided eye in the Superagglutination test.

**Fig. 4:**
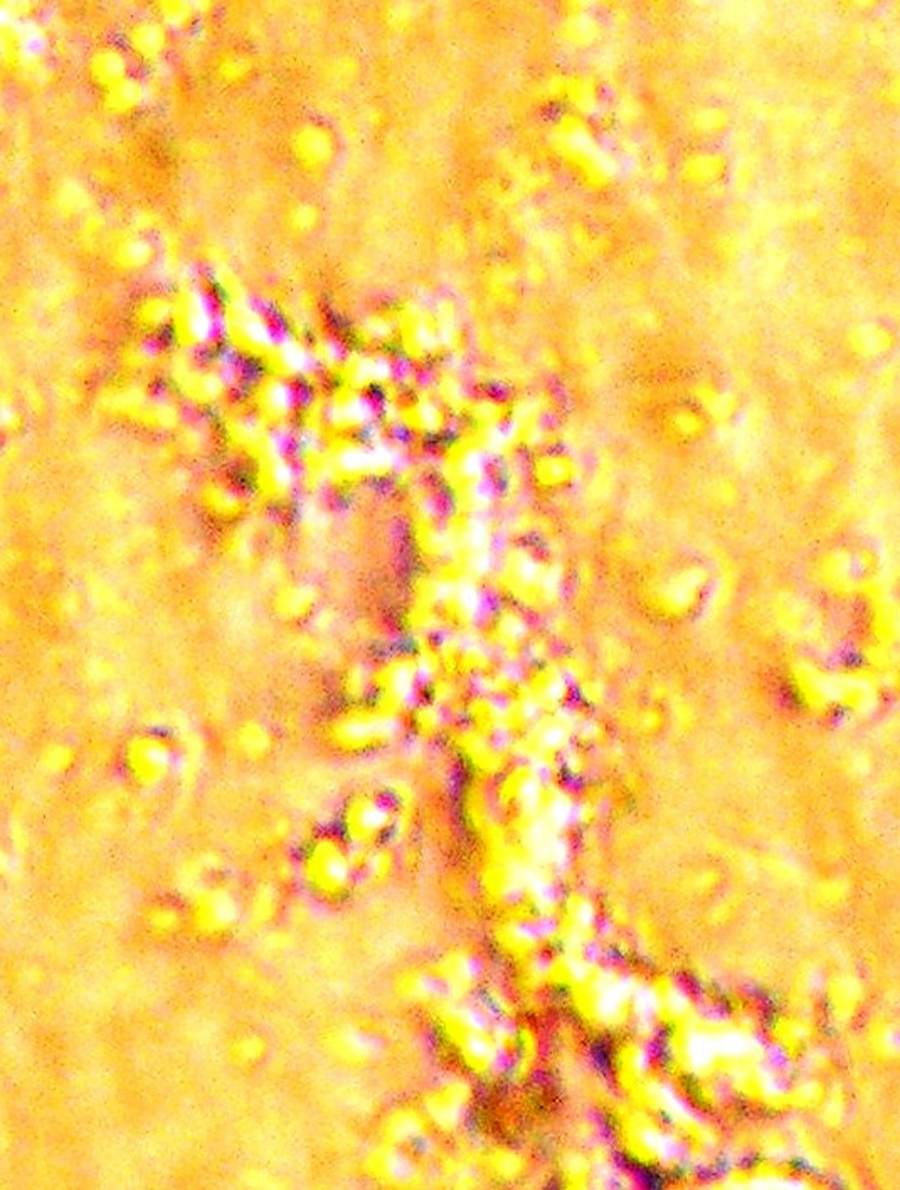
Microscopic view of the slide (low power) in RBPT.

**Fig. 5:**
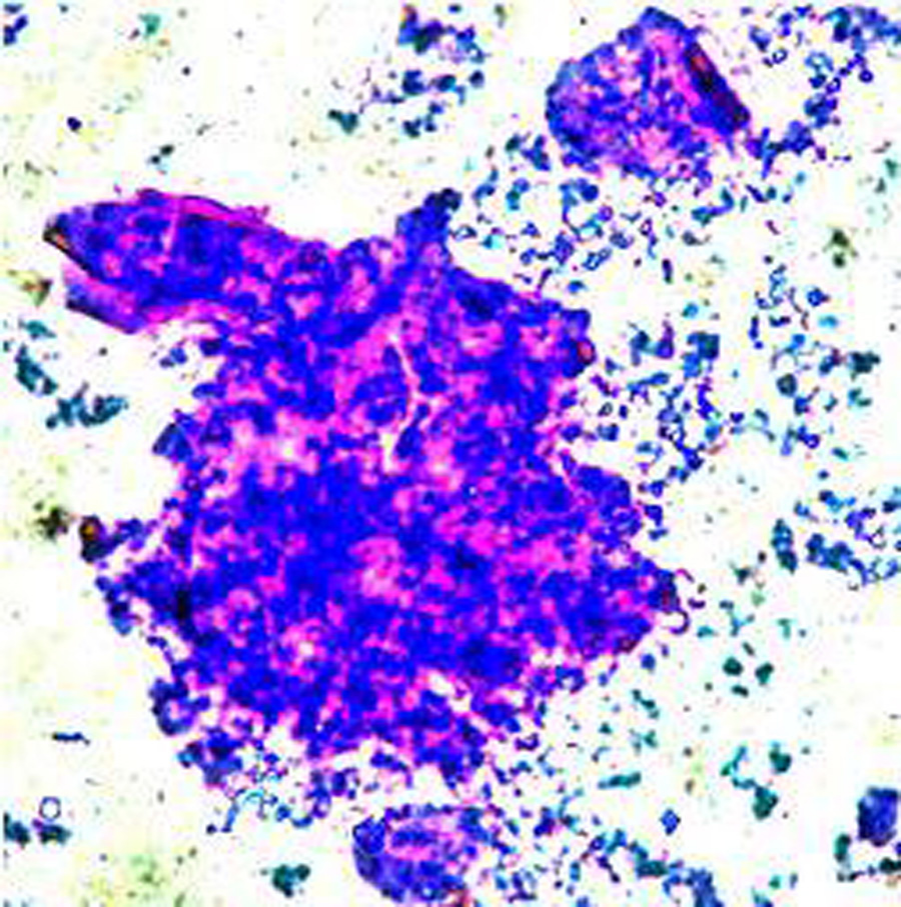
Microscopic view of the slide (low power) in the Superagglutination test.

In case of the Superagglutination test (Fig. 3, Fig. 5), the clumps on the slide had both blue and pink color. When the slide was viewed under the low power of a light microscope, the true agglutinate could be very easily differentiated into two parts, the antibodies were blue in color due to the Coomassie Blue dye and the antigen was pink in color due to the Rose Bengal dye. Clumps of a larger size (1.8 times bigger) were formed in Superagglutination compared to RBPT. The difference in clump size was significant (*p* < 0.05) (Fig. 3, Fig. 5). The Superagglutination test detected more positive samples than ELISA (16.5%), CFT (14.5%), RBPT (6%) and STAT (6%) and showed a sensitivity of 95.88% and a specificity of 89.32%. The positive predictive value (PPV) of this test was found to be 89.42% and Negative Predictive Value (NPV) was 95.83% (Table 3). The statistical agreement between the Superagglutination test and RBPT was found to be almost perfect, whereas the agreement between the Superagglutination test and other tests was found to be substantial (Table 4).

Superagglutination could identify all the sera giving false negative results as well as the sera giving false positive results by RBPT, STAT and ELISA, respectively. It could detect all the positive samples that went undetected by other tests (i.e., false negative) including sera tested negative by a combination of RBPT, STAT and ELISA, negative by a combination of RBPT and STAT, negative by both STAT and ELISA and those negative by both RBPT and ELISA, respectively. No false positives results were obtained in case of the Superagglutination when sera of brucellosis - free animals were tested and results were compared with those of RBPT, STAT, and ELISA.

Our simple and easy modifications very significantly enhance the sensitivity of the agglutination test because of formation of compact and larger clumps without compromising the specificity and hence minimize the chances of false negative results occurring due to low titer of antibody in the serum. The staining of antibody in the serum/plasma eliminates the false positive results arising due to non-specific aggregates of antigen particles caused by improper mixing mistaken for an agglutinate. The real agglutinates will be of two colors due to the colored antigen and the stained antibody whereas aggregates of antigen particles alone or antibody alone will be of one color only.

Our new modifications in the agglutination test do not require any extra equipment or skill and they do not cost much since only a few microlitres of the stain, the diluted biotinylated antiglobulin and diluted Avidin are required for each test. Furthermore, the additional steps do not require more than five minutes. The antiglobulin of IgG isotype, being bivalent, can give two-fold enhancement in agglutination whereas, the antiglobulin of IgM isotype, due to its ten binding sites, can give up to ten-fold enhancement in agglutination. The binding of Avidin with Biotin on the biotinylated antiglobulin makes the clumps up to 4 times larger and compact due to four binding sites for biotin on each avidin molecule and hence they are easily detectable.

The novel Superagglutination test could detect more positive samples than RBPT, STAT, iELISA and CFT in the diagnosis of bovine brucellosis. The highest sensitivity of all the tests observed with Superagglutination test in our study can be attributed to the fact that the number of false negative results was less with the Superagglutination test when compared to the other tests. The specificity of the Superagglutination test was found to be higher than that of RBPT and STAT. In the case of the Superagglutination test, the two colored true agglutinates could be very easily differentiated from non-specific one colored aggregates under the low power of a light microscope. Each agglutinate had both the blue and the pink color, which aided in the differentiation of the true agglutinates from the non-specific aggregates of the antigen of pink color only. The antigen and antibodies which did not participate in agglutination reaction could be viewed under the microscope as aggregates of either blue or pink particles alone lying separately.

The highest agreement with RBPT combined with the higher specificity and sensitivity of Superagglutination test ensures that it can serve as a more efficient screening test than RBPT. The Superagglutination test had a higher sensitivity and a negative predictive value than the other serodiagnostic tests like RBPT, STAT, ELISA and CFT. Its specificity and PPV were found to be better than RBPT and STAT. The test can be used in the pen side diagnosis of bovine brucellosis with better results than the RBPT which is routinely used as a pen side test for brucellosis. ELISA is not a cost effective test when screening has to be performed on herds with a large number of animals. In such situations, Superagglutination test can offer an advantage of increased sensitivity of screening compared to RBPT and STAT.

The Superagglutination test showed the highest sensitivity of all the tests (95.88%) which can be attributed to the lesser number of false negative results obtained with Superagglutination test compared to the other tests. Interestingly, the agglutinate in the case of Superagglutination test had two discernible constituents, the blue antibodies and the pink antigen. which aided in the differentiation of the true agglutinates from aggregates of particles. This has not been reported earlier by any other researcher. The results obtained in our study suggest that Superagglutination test can be used as a screening test in the diagnosis of bovine brucellosis. The innovative concepts behind our new modifications to the agglutination test are applicable not only to brucellosis but to other infectious diseases of animals and humans wherever agglutination test is applicable.

## Figures and Tables

**Table 1:** Number of positive and negative samples in each of the test conducted.

Test conducted	Number of samples		
	Positive	Negative	Total
Superagglutination	104	96	200
RBPT	97	103	200
STAT	119	81	200
iELISA	75	125	200
CFT	86	114	200

**Table 2:** Difference between Superagglutination test and other serological tests regarding positive and negative samples identified.

	Difference with the other tests							
	Positive samples				Negative samples			
	RBPT	STAT	iELISA	CFT	RBPT	STAT	iELISA	CFT
Superagglutination test	+12	+12	+33	+29	−03	−25	−04	−09

+, more number detected by Superagglutination test; − less number detected by Superagglutination test.

**Table 3:** Samples found negative with one test but positive by other serologic tests.

Sample No.	RBPT	STAT	Superagglutination	ELISA
**RBPT−ve**				
1/21(M1), M2, M4, M5, M7, M8	Negative	Negative	**Positive**	Negative
M9, 2-S	Negative	Negative	**Positive**	**Positive**
M10, M12, M13, M15, A2, 8-P, 10-P	Negative	Negative	**Positive**	Negative
M17, M18, M19, M20, J-6, J-19	Negative	Negative	Negative	**Positive**
3-P	Negative	**1:40**	**Positive**	Negative
5-P	Negative	**1:40**	Negative	**Positive**
7-P, 9-P	Negative	Negative	**Positive**	Negative
J-4	Negative	Negative	**Positive**	**Positive**
**STAT−ve**				
6, 136P/J26, M3, M25, M30	**Positive**	Negative	**Positive**	Negative
J-15, 07-1258, 07-1222, 47	**Positive**	Negative	**Positive**	**Positive**
1/21(M1), M2, M4, M5, M7, M8, M10, M12, M13, M15, M24, M28, A2, 8-P, 10-P	Negative	Negative	**Positive**	Negative
M9, 2-S	Negative	Negative	**Positive**	**Positive**
M17, M19, M20, J-6, J-19	Negative	Negative	Negative	**Positive**
**ELISA−ve**				
136P/J26, M3	**Positive**	Negative	**Positive**	Negative
1/21(M1), M2, M4, M5, M7, M8, M10, M12, M13, M15, A2, 8-P, 10-P	Negative	Negative	**Positive**	Negative
3-P	Negative	**1:40**	**Positive**	Negative
7-P, 9-P	Negative	Negative	**Positive**	Negative

The bold signifies that this result is different from that obtained with another test on the same sample.

**Table 4:** Samples found positive with one test but negative by other serologic tests.

Sample no.	RBPT	STAT	Superagglutination	ELISA
**RBPT**+**ve**				
4-C, 5-C	Positive	**Negative**	Positive	Positive
136P/J26	Positive	**Negative**	Positive	**Negative**
J17	Positive	1:40	Positive	Positive
J18	Positive	**Negative**	Positive	Positive
07-1258	Positive	**Negative**	Positive	Positive
J-7	Positive	**Negative**	**Negative**	Positive
**STAT**+**ve**				
4-C, 5-C	Positive	Positive	**Negative**	Positive

The bold signifies that this result is different from that obtained with another test on the same sample.
